# RAGE acts as an oncogenic role and promotes the metastasis of human lung cancer

**DOI:** 10.1038/s41419-020-2432-1

**Published:** 2020-04-23

**Authors:** Mei-Chih Chen, Kun-Chieh Chen, Gee-Chen Chang, Ho Lin, Chun-Chi Wu, Wei-Hsiang Kao, Chieh-Lin Jerry Teng, Shih-Lan Hsu, Tsung-Ying Yang

**Affiliations:** 1 Translational Cell Therapy Center, Department of Medical Research, China Medical University Hospital, Taiwan, No. 2, Yude Road, North District, Taichung City, 40447 Taiwan; 2 Department of Nursing, Asia University, Taiwan, No. 500, Lioufeng Rd., Wufeng Taichung City, 41354 Taiwan; 3 Division of Chest Medicine, Department of Internal Medicine, Taichung Veterans General Hospital, Taiwan, No. 1650, Taiwan Boulevard Sect. 4, Taichung, 40705 Taiwan; 4 Department of Life Sciences, National Chung Hsing University, Taiwan, No. 145, Xingda Rd., South Dist., Taichung City, 402 Taiwan; 5 Institute of Medicine, Chung Shan Medical University, Taiwan, No. 110, Sec.1, Jianguo N.Rd., Taichung City, 40201 Taiwan; 6 Division of Hematology/Medical Oncology, Department of Medicine, Taichung Veterans General Hospital, Taiwan, No. 1650, Taiwan Boulevard Sect. 4, Taichung, 40705 Taiwan; 7 Department of Life Science, Tunghai University, Taichung City, Taiwan, No. 181, Sect. 3, Taichung Port Road, Taichung City, 40704 Taiwan; 8 School of Medicine, Chung Shan Medical University, Taichung, Taiwan, No.110, Sec. 1, Jianguo, N. Rd., Taichung City, 40201 Taiwan; 9 Department of Medical Research, Taichung Veterans General Hospital, Taiwan, No. 1650, Taiwan Boulevard Sect. 4, Taichung, 40705 Taiwan; 10 Faculty of Medicine, School of Medicine. National Yang-Ming University, Taiwan, No. 155, Sec.2, Linong St., Taipei, 112 Taiwan

**Keywords:** Non-small-cell lung cancer, Non-small-cell lung cancer

## Abstract

RAGE (receptor for advanced glycation end-product) is thought to be associated with metastasis and poor prognosis of various types of cancer. However, RAGE is constitutively expressed in the normal lung and down-regulated in cancerous lung, while the opposite evidence shows that RAGE-mediated signaling contributes to the tumorigenesis of lung cancer. Therefore, the role of RAGE in lung cancer progression is still unclear to be further investigated. In this study, RAGE-overexpressed stable clones of human lung cancer A549 cells and two local lung adenocarcinoma cell lines CL1-0 and CL1-5 were utilized to verify the effect of RAGE on lung cancer cells while the in vivo xenograft animal model was further performed to evaluate the role of RAGE in the progression of lung cancer. The growth of A549 cells was inhibited by RAGE overexpression. p53-dependent p21^CIP1^ expression contributed to RAGE-induced growth inhibition by suppressing CDK2 kinase activity and retinoblastoma protein (RB) phosphorylation in vitro. On the other hand, RAGE overexpression promoted migration, invasion, and mesenchymal features of lung adenocarcinoma cells through ERK signaling. Furthermore, an in vivo xenograft experiment indicated that RAGE promoted the metastasis of lung cancer cells with p21^CIP1^ up-regulation, ERK activation, and the changes of EMT markers. Regarding to the involvement of tumor-associated macrophage (TAM) in the microenvironment, we monitored the expressions of TAM markers including CD68 and CD163 as well as angiogenesis marker CD31 in xenograft slice. The data showed that RAGE might induce the accumulation of TAM in lung cancer cells and further accelerate the in vivo tumor growth. In summary, our study provides evidence indicating the distinct in vitro and in vivo effects of RAGE and related mechanisms on tumor growth and metastasis, which shed light on the oncogenic role of RAGE in lung cancer.

## Introduction

RAGE, a member of the immunoglobulin superfamily, is a multi-ligand receptor interacting with distinct molecules^1^. It is implicated in physiological homeostasis, development, inflammation, and several diseases^2–4^. In addition, both RAGE and its ligands are increased and associated with metastasis and poor prognosis in various types of malignant tumors, including prostate, gastric, breast, and colon cancers^5–8^. Contradictory to the findings in most cancers, RAGE is constitutively expressed in normal lung and appears to be downregulated in human lung cancer^9^. The downregulation of RAGE has been thought to be positively correlated with lung tumor growth and invasiveness^9–11^, therefore, it is considered a diagnostic marker of lung cancer^12^. However, Hsieh et al. reported the abundant expression of RAGE, as well as its ligand S100A6, in human lung tumor tissues and suggested a positive role of RAGE-mediated signals in the development of lung cancer^13^. In addition, Taguchi et al. demonstrated that the blockade of RAGE-amphoterin signaling decreases the growth and metastasis of lung cancer in a xenograft model^14^. Moreover, RAGE-ligand high-mobility group box protein 1 (HMGB1) promotes the proliferation and anti-apoptosis of Lewis lung cancer cells through RAGE and toll-like receptor 4 (TLR4)-dependent signals^15^. RAGE has been shown to be highly expressed in the basal membrane of type 1 alveolar epithelial (AT1) cells in normal lung^16^. Oczypok et al. claimed that even though RAGE expression is likely to increase in the lung tumor cells, the bronchogenic tumors appear to have less RAGE than healthy lungs with large numbers of AT1 cells, because the predominant cell-type in lung cancers is the bronchial epithelial cell, which does not typically express RAGE^17^. Accordingly, the role of RAGE in lung cancer progression is still unclear. Further insights into the effects and mechanisms behind the expression of RAGE in lung tumorigenesis are greatly needed.

In this study, we established RAGE-overexpressed stable clones of human lung cancer cells to evaluate the role of RAGE in the tumorigenesis of lung cancer. We found that RAGE increased a p53-dependent p21^CIP1^ expression, which inhibited cell growth through a CDK2 inactivation manner. However, RAGE on the other hand promoted cell migration and invasion through ERK1/2–Snail/Slug/Twist–E-cadherin cascades. Furthermore, the xenograft animal model provided evidence suggesting an oncogenic effect of RAGE through modulating the tumor microenvironment (TME) of lung cancer.

## Results

### RAGE inhibits lung cancer cell growth

**Fig. 1:**
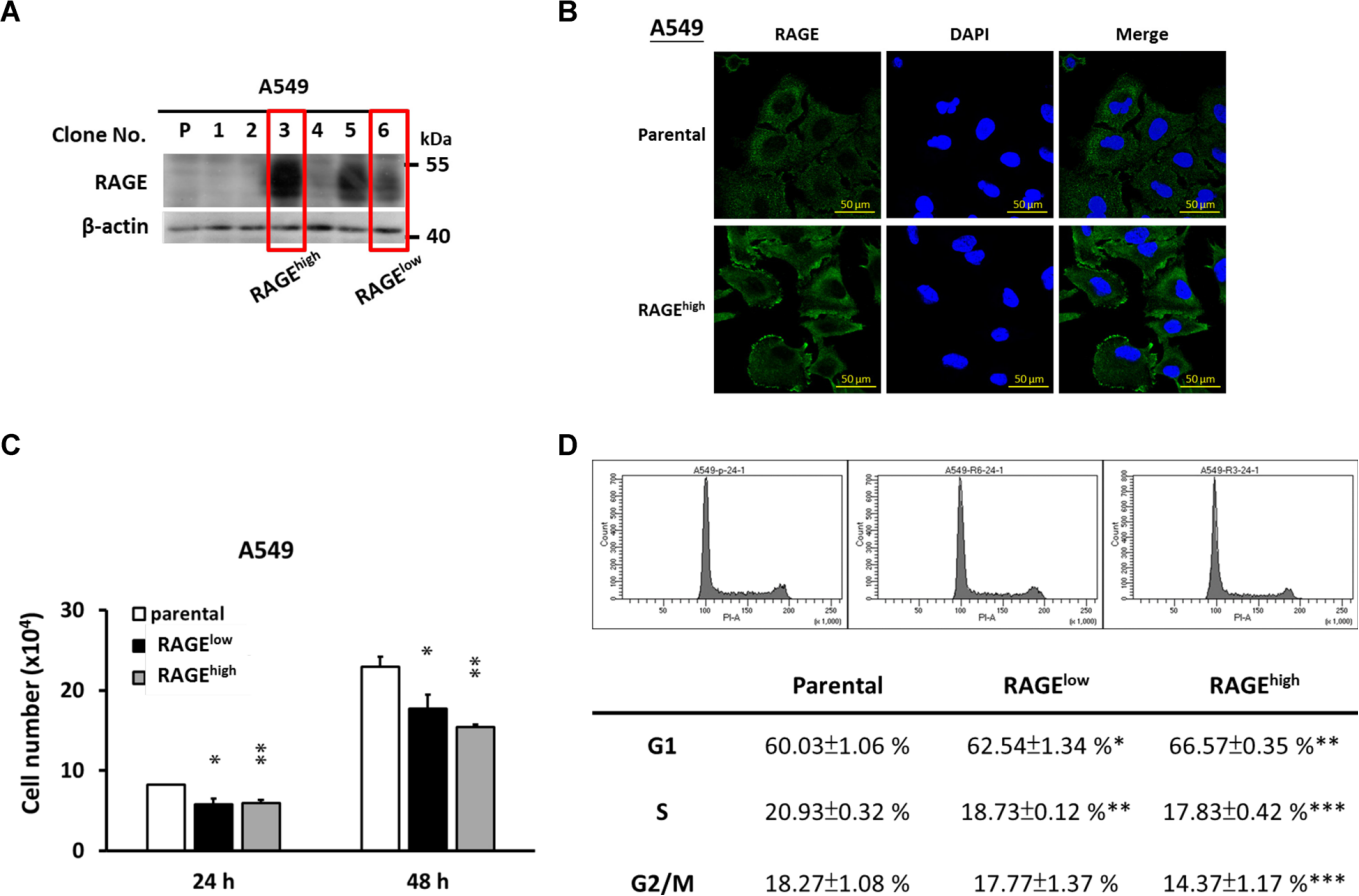
The growth inhibition of RAGE-overexpressed A549 cells. An expression vector containing human RAGE open reading frame (pUNO1-hAGER, InvivoGene) or empty-vector (pUNO1-mcs) was transfected into A549 cells following by continuous screening for 8 weeks. **a** Western blotting was performed to evaluate the protein levels of RAGE in different subclones. β-actin served as the internal control. Two subclones were selected and renamed as RAGE^low^ and RAGE^high^ for future examination. **b** The intracellular distributions of RAGE (green signal) in A549 stable cell lines were monitored by immunocytochemistry, and DAPI was the marker of nucleus (blue signal). **c** 5 × 10^3^ cells/well were seeded into 24-well culture plates and cultured with complete medium. The cell number was counting every 24 h and the cell growth of different subclones of A549 cells was shown. **d** Cell cycle distribution of A549 stable cell lines was analyzed by flow cytometry after propidium iodide (PI) staining. The quantitative data from three repeated samples was also provided. Parental cell line was used as the control group. *p < 0.05; **p < 0.01; ***p < 0.001 compared with control group.

RAGE overexpressed A549 subclones were prepared as described in the Materials and methods section. Two of them were randomly selected and renamed as clone RAGE^low^ and RAGE^high^ for subsequent experiments. The protein level and subcellular distribution of RAGE is shown in Fig. 1a, b. RAGE protein was more abundant and concentrated at the cell edge in RAGE overexpressed A549 cells. To address the role of RAGE in the fate of the A549 cells, the cell growth and cell cycle distribution of different subclones were evaluated. As shown in Fig. 1c, the overexpression of RAGE significantly inhibited the growth of A549 cells. In addition, the percentage of cells undergoing the G1 phase was increased in RAGE-overexpressed subclones compared to their parental cells (Fig. 1d).

### RAGE upregulates p53 and p21CIP1 and inhibits CDK2 activity

**Fig. 2:**
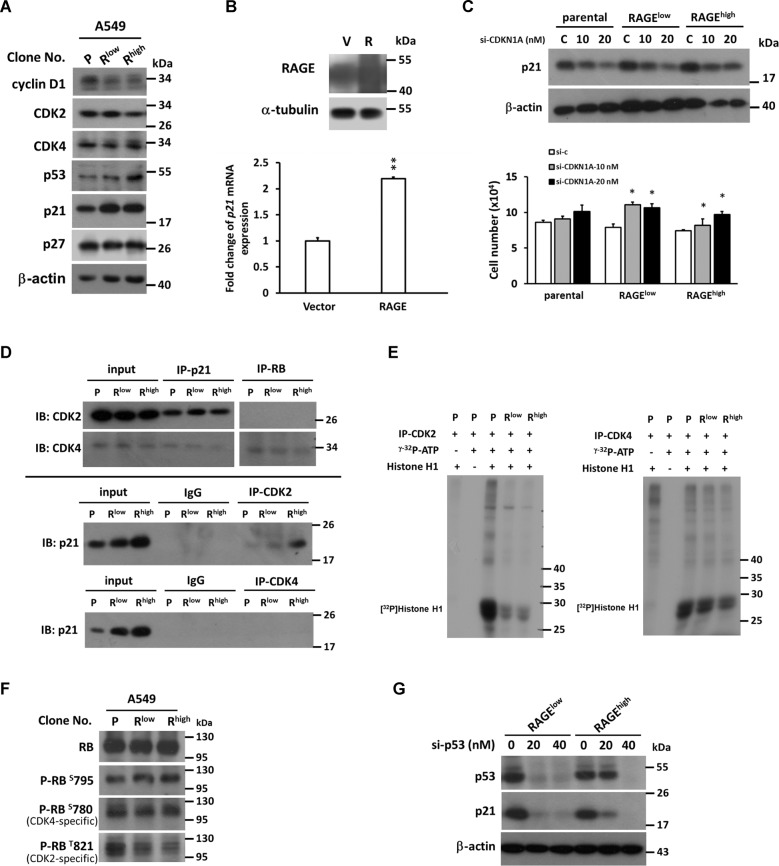
The importance of p21CIP1 up-regulation in RAGE-overexpressed A549 cells. **a** Western blotting analysis of cell-cycle-related regulators in RAGE-overexpressed subclones of A549 was shown; **b** p21^CIP1^ mRNA expression was measured by quantitative real-time PCR in A549 cells after transient transfection of RAGE; **c** A549 subclones were transfected with control siRNA or siRNA against CDKN1A (p21^CIP1^). Cell growth was measured to evaluate the effect of p21^CIP1^ knockdown in RAGE-overexpressed A549 subclones; **d** protein interactions between p21^CIP1^-CDK2/4 and RB-CDK2/4 were evaluated by immunoprecipitation; **e** the kinase activities of CDK2 or CDK4 were detected by in vitro kinase assay while Histone H1 was used as the specific substrate; **f** the levels of CDK2-specific T821 residue phosphorylation of RB(P-RB^T821^) and CDK4-specific S780 residue (P-RBS^780^) were detected by western blotting analysis; **g** p53 was knockdown by transfection of siRNA in A549 subclones and the protein levels of p21^CIP1^ and p53 were measured. β-actin served as the internal control. P stands for parental and R^low^/R^high^ stands for different clones of the cells. *p < 0.05; **p < 0.01 compared to parental cells.

Next, the expression pattern of cell cycle regulators was analyzed, and p53 and p21^CIP1^ levels were significantly increased in RAGE-overexpressed subclones (Fig. 2a). Additionally, mRNA expression of p21^CIP1^ was increased in RAGE-overexpressed cells (Fig. 2b). To address the role of p21^CIP1^ in RAGE-mediated growth inhibition, knockdown of p21^CIP1^ was conducted and RAGE-induced growth inhibition was reversed (Fig. 2c) correspondingly. The result was consistent with knockdown of p21^CIP1^ in A459 cells transiently overexpressing RAGE; SI 1: Fig. S1). To define the mechanistic action of p21^CIP1^, immunoprecipitation was carried out to examine the protein interaction between p21^CIP1^ and CDKs responding to G1 phase (CDK2 and CDK4). In Fig. 2d, the interaction of p21^CIP1^ and CDK2 was increased in RAGE-overexpressed cells, while the interaction between p21^CIP1^ and CDK4 was not altered. Data from kinase activity assay revealed that CDK2 activity significantly reduced in RAGE-overexpressed subclones (Fig. 2e). Activated CDK/cyclin complexes can phosphorylate specific amino acid residues of RB^17–19^. In Fig. 2f, the phosphorylation of CDK2-specific T821 residues of RB (P-RBT821) was significantly decreased in RAGE-overexpressed cells. Moreover, to address whether RAGE-induced p21^CIP1^ upregulation is affected by p53 status, p53 knockdown was performed and p21^CIP1^ levels were further detected. As shown in Fig. 2g, the p21^CIP1^ level in RAGE-overexpressed cells was declined after p53 knockdown, suggesting RAGE-induced p21^CIP1^ upregulation was p53-dependent.

### RAGE downregulates E-cadherin expression through ERK1/2-dependent Snail/Slug/Twist pathway and enhances lung cancer cell migration

**Fig. 3:**
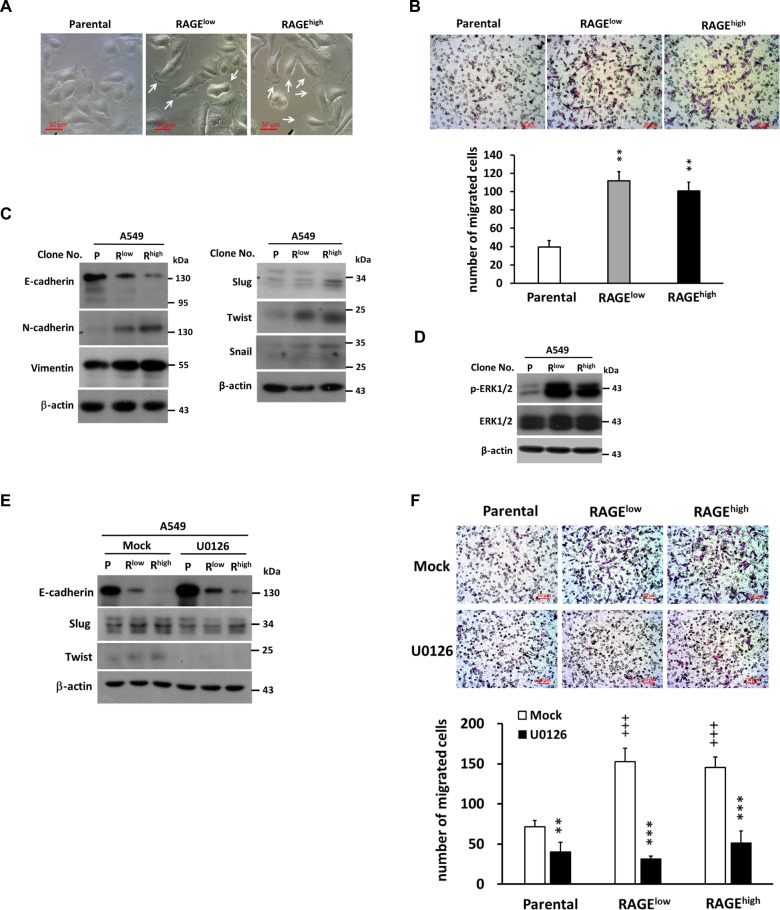
RAGE-induced migration of A549 cells is mediated through ERK activation. **a** The cell morphology was recorded and increased lamellipodia were observed in RAGE-overexpressed subclones (white arrows); **b** The migration ability was evaluated by Transwell assay. The upper insert contained cells suspended in serum-free medium (5 × 10^4^ cells/ 200 μl), while the lower compartment contained complete medium with 10% FBS. After incubation for 6 h, filters were fixed with methanol followed by Giemsa staining. The migrated cells were counted in three random microscopic field images. Quantitative results from three independent experiments were shown below; **c** Western blotting was performed to detect the expression of EMT markers and **d** phosphorylated levels of ERK protein in A549 subclones with β-actin served as the internal control. **e** ERK inhibitor (U0126, 20 μM) was added into the culture medium for 24 h, cells were harvest following by protein extraction. The effect of ERK inhibition on EMT marker E-cadherin and migration related proteins were evaluated. **f** The effect of ERK inhibition on RAGE-promoted migration of A549 cells was evaluated by Transwell assay followed by Giemsa staining. The quantitative results were also shown. β-actin served as the internal control. P stands for parental and R^low^/R^high^ stands for different clones of the cells. **p < 0.01; ***p < 0.001 compared to Mock group; ^+++^*p* < 0.001 compared to parental cells.

Lamellipodium plays a decisive role in cell motility and migration. Increased lamellipodia formation was observed in RAGE-overexpressed cells (Fig. 3a), revealing that overexpressed RAGE might facilitate cell migratory ability. Accordingly, a transwell migration assay was assessed and, as shown in Fig. 3b, RAGE significantly promoted the migratory activity of A549 cells. This event was further confirmed by real-time, live cell imaging of the wound healing assay (SI 2: Video 1, SI 3: Video 2. and SI 4: Fig. S2).

EMT is critical for cancer metastasis. Therefore, the effect of RAGE on EMT-associated molecules was evaluated. The protein level of E-cadherin was decreased, while N-cadherin and vimentin were increased in RAGE-overexpressed subclones (Fig. 3c). In addition, the levels of Snail, Slug, and Twist were increased in RAGE-overexpressed cells.

ERK signaling was reported to participate in EMT transcription factors mediated downregulation of E-cadherin in cancers^20–22^. In Fig. 3d, the level of phosphorylated-ERK1/2 was elevated in RAGE-overexpressed A549 cells. To characterize the role of ERK1/2 in RAGE-mediated regulation of EMT-associated molecules as well as migration promotion, an ERK1/2 inhibitor U0126 was used to block ERK1/2 signaling. As depicted in Fig. 3e, the expression pattern of Slug, Twist, and E-cadherin was reversed after ERK1/2 inhibition in RAGE-overexpressed cells. Furthermore, RAGE-promoted migratory ability was significantly obstructed while ERK signaling was inhibited (Fig. 3f).

### The comparison of RAGE expression in CL1-0 and CL1-5 lung adenocarcinoma cells

**Fig. 4:**
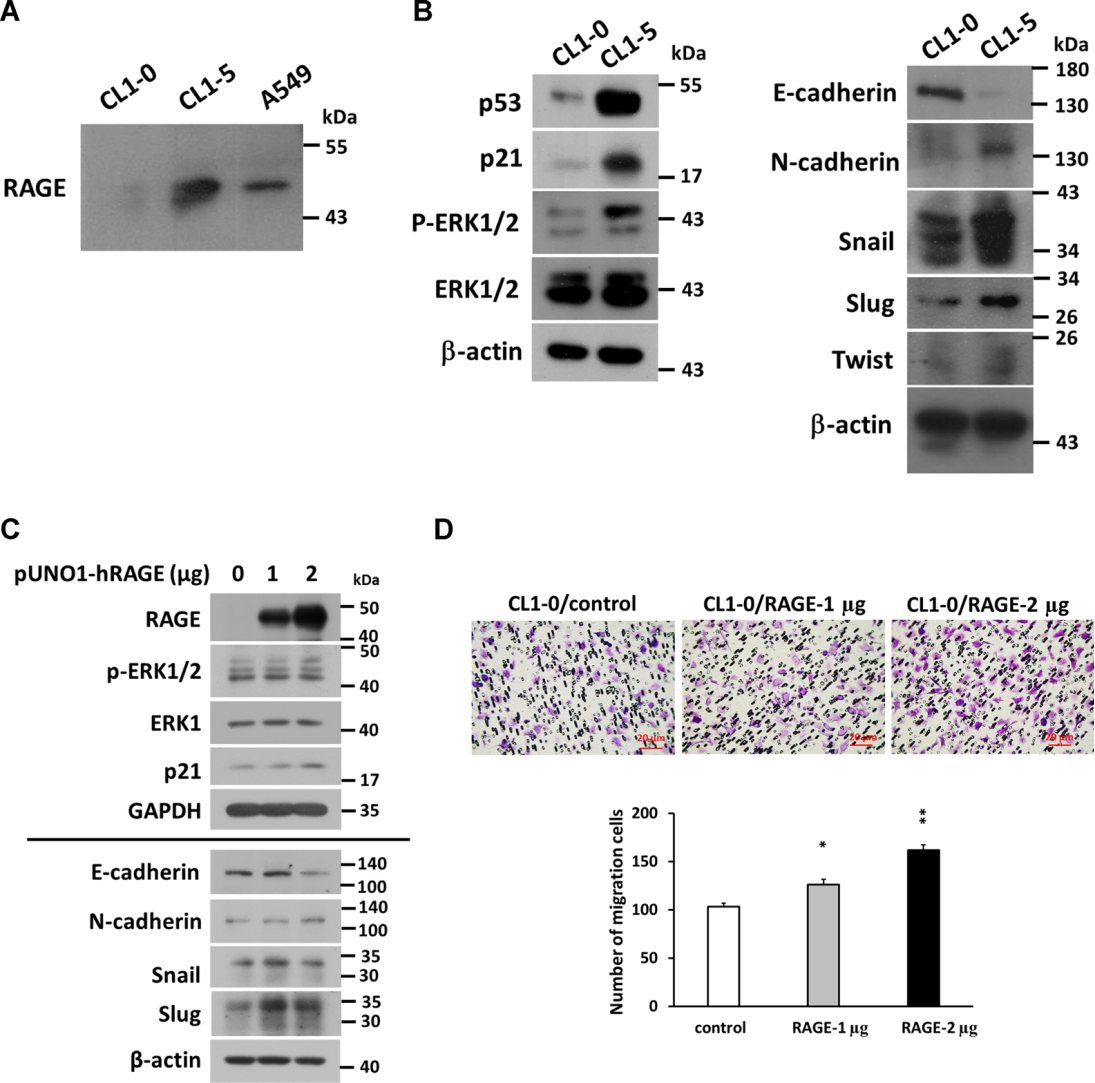
The expression survey of RAGE and related proteins in CL series cells. **a** The RAGE level in CL1-0 and CL1-5 cells was evaluated by western blotting, **b** the protein levels of ERK, p-ERK, p53, p21^CIP1^, and EMT-related proteins were compared between CL1-0 and CL1-5 cells. β-actin served as the internal control. **c** CL1-0 cells were transfected with human RAGE, and the protein levels of RAGE, ERK, p-ERK, p21^CIP1^, EMT markers, Snail, and Slug were detected by western blotting. **d** The migration ability was evaluated by performing Transwell assay after RAGE overexpressed in CL1-0 cells. *p < 0.05; **p < 0.01; ***p < 0.001 compared with control group.

In addition to A549 cells, two local lung adenocarcinoma cell lines, CL1-0 and CL1-5, were utilized to verify the effect of RAGE on the migration of lung cancer cells. CL1-5 cells have a higher migration and invasion ability than CL1-0 cells^18^. However, they grew slower than CL1-0 cells (SI 5: Fig. S3), and we found that RAGE level in CL1-5 was higher than in CL1-0 cells (Fig. 4a). Additionally, protein levels of p21^CIP1^, p-ERK1/2, and EMT-related molecules—including N-cadherin, Snail, Slug, and Twist —in CL1-5 were higher, while E-cadherin was lower than in CL1-0 cells (Fig. 4b). In CL1-0 cells, transient transfection of RAGE resulted in increased levels of RAGE, p21^CIP1^ and p-ERK1/2, whereas EMT-related markers showed a similar performance as found in RAGE-overexpressed A549 cells (Fig. 4c). Furthermore, RAGE facilitated the migration of CL1-0 cells (Fig. 4d), demonstrating a positive correlation between RAGE and cell migration in other lung adenocarcinoma cells besides A549 cells.

### RAGE promotes metastasis in a xenograft animal model

**Fig. 5:**
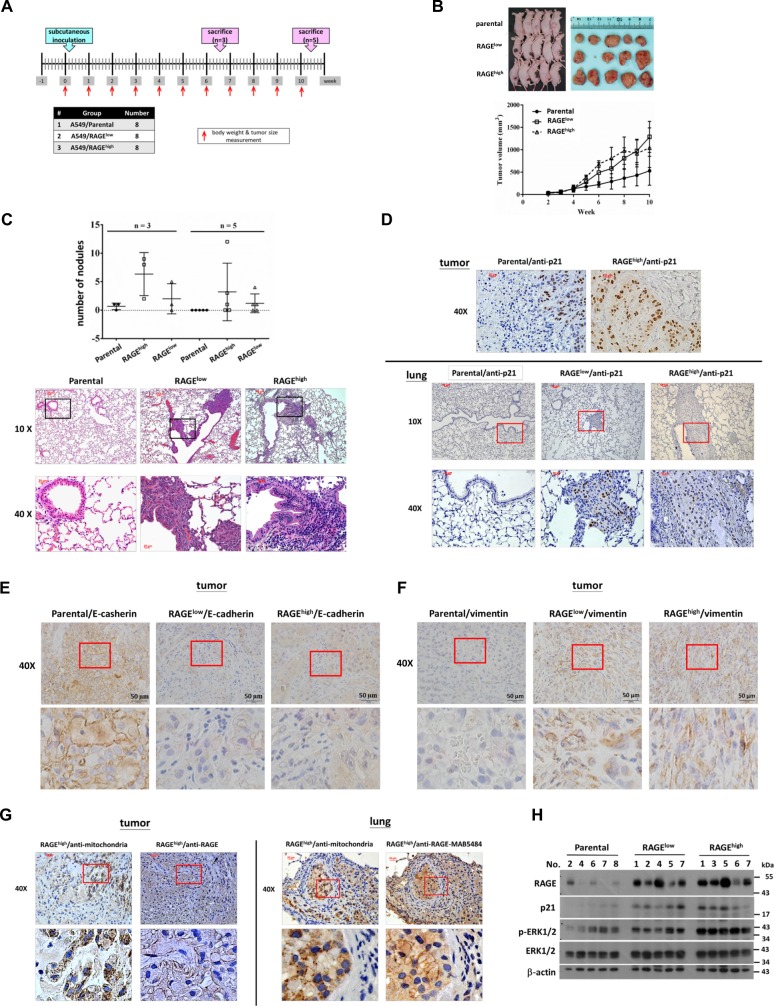
The effects of RAGE on tumorigenesis and metastasis in xenograft model. **a** The experimental design and procedures. **b** A549 subclones were injected subcutaneously into the right low flank of mice. The tumor volume was measured every week and the tumor growth curves were recorded. At the end of the experiment, tumors were excised from the mice. **c** The number of visible metastatic nodules on the surface of the lungs was calculated. The morphology of metastatic lung tumor sections was shown using H&E staining. The inset images were magnified in the lower panels. **d** The A549 subclone-derived tumors and mice lung tissues were subjected to IHC staining for p21^CIP1^ expression and visualized using the ABC system. **e** The IHC staining for E-cadherin expression in A549 subclones-derived tumors was performed, and the images were visualized using the ABC system. **f** The A549 subclones-derived tumors were subjected to IHC staining for vimentin expression and visualized using the ABC system. **g** The IHC staining for RAGE expression in the xenograft tumor and lung tissue sections was performed, and the images were visualized using the ABC system. Human-mitochondria-specific antibody (ab92824, Abcam, Cambridge, UK.) was used to label human cells, and the images were shown. The inset images were magnified in the lower panels. **h** The protein levels of RAGE, p21^CIP1^, phospho-ERK1/2, and ERK1/2 in xenografts were measured using western blotting, and β-actin served as the internal control. *p < 0.05, **p < 0.01 and ***p < 0.001 compared to the parental group.

To confirm the effect of RAGE on lung cancer metastasis in vivo, A549 cells of each of the subclones were subcutaneously injected into male nude mice to establish a xenograft animal model (Fig. 5a). The body weights showed no significant differences among each group during the experimental period (SI 6: Fig. S4). However, the average of tumor size was larger in RAGE groups than in the parental group after the 5th week (Fig. 5b). The lungs and subcutaneous tumors were collected, and the visible lung metastatic nodules were counted (SI 7: Fig. S5). As shown in Fig. 5c, the number of visible metastatic nodules in lung was higher in RAGE-overexpressed groups, and the hematoxylin and eosin (H&E) stain of lung tissue specimens also revealed the presence of metastatic tumors in RAGE groups. These results indicated that RAGE promotes lung cancer metastasis in vivo. An immunohistochemistry (IHC) assay indicated that p21^CIP1^ was highly expressed both in metastatic nodules and in inoculated tumors of RAGE groups (Fig. 5d). The expression of EMT markers including E-cadherin and vimentin were consistent with the results of the in vitro experiments (Fig. 5e, f). Additionally, RAGE expression in inoculated tumors and lung metastatic nodules was detected by IHC, while human-mitochondria-specific antibody (ab92824, Abcam) was used to label human-derived lung cancer cells (Fig. 5g). Consistent with in vitro data, protein levels of RAGE, p21^CIP1^, and phosphorylated ERK1/2 all increased in inoculated tumors of RAGE groups in comparison with the tumors of the parental group (Fig. 5h).

### RAGE modulates the tumor microenvironment in a xenograft animal model

**Fig. 6:**
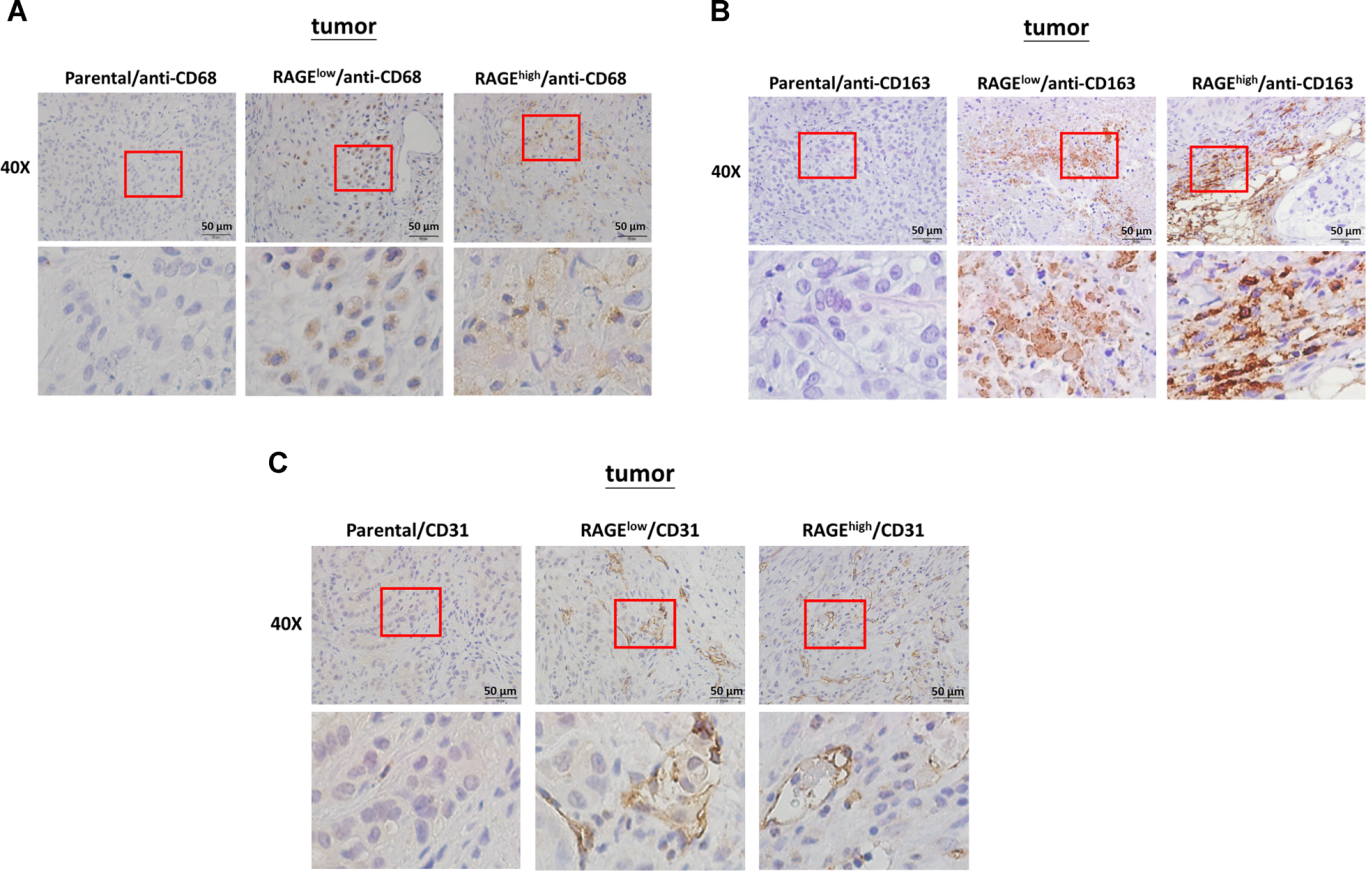
The effects of RAGE on TAM infiltration in xenograft model. **a** The A549 subclones-derived tumors were subjected to IHC staining for TAM marker CD68 expression. **b** The IHC staining for M2 macrophage marker CD163 expression in A549 subclones-derived tumors were performed. **c** The IHC staining for endothelial target CD31 in A549 subclones-derived tumors, and all the images were visualized using the ABC system.

Since the in vivo data of tumor growth were inconsistent with in vitro, which factors promoted the in vivo tumor growth in RAGE-overexpressed groups were further evaluated. Tumor-associated macrophages (TAMs) in tumor microenvironment (TME) are responsible for the development and progression of tumor^19–21^. To evaluate the effect of RAGE on the recruitment of TAMs in vivo, the expression profile of TAM markers CD68 and CD163^22–24^ in the inoculated tumors was evaluated by IHC assay. The higher expression levels of both CD68 and CD163 were observed in RAGE-overexpressed groups compared with in parental group (Fig. 6a, b). In addition to TAM markers, the expression of the endothelial marker CD31^25,26^ in tumor sections was also identified to evaluate the contribution of RAGE on the beneficial microenvironment for angiogenesis. The results in Fig. 6c showed that CD31 was highly expressed in RAGE-overexpressed groups compared to parental group.

## Discussion

In this study, we evaluated the role of RAGE in the tumorigenesis of lung adenocarcinoma. The in vitro results indicated that RAGE overexpression retarded cell growth, increased G1 phase distribution, and promoted EMT in lung adenocarcinoma cells. p21^CIP1^ participated in RAGE-induced growth inhibition and G1 retardation through inhibiting CDK2 kinase activity and targeting specific RB phosphorylation. On the other hand, RAGE enhanced migration ability of lung cancer cells and promoted EMT progression through an ERK1/2-related pathway. Furthermore, the results from the tumor xenograft mice experiment provided solid evidence that RAGE promotes lung cancer metastasis and provides a beneficial TME for cancer progression.

RAGE is expressed more abundantly in mature lung than in other tissues, indicating its specific role in the lung^27^. In addition to a receptor, RAGE appears to be a differentiation marker and participates adherence, spreading, and gas exchange of alveolar type I cells in normal lung^27,28^. Downregulation of RAGE is thought to trigger lung tumor formation because RAGE ligand amphoterin, which is highly expressed in the lung, mediates cell differentiation via RAGE^9,11^. Here, we provide evidence that RAGE inhibits cell growth and retards the cell cycle progression through a p53-dependent p21^CIP1^ upregulation (Fig. 2). This finding might be connected to the normal function of RAGE in alveolar epithelial cells, in which RAGE takes part in maintaining a differentiation appearance and depressing cell growth through maintaining p21^CIP1^ performance at a high level. Therefore, in the early stage of tumorigenesis, the reduction of RAGE might destroy its stimulating effect on p21^CIP1^ expression and function. This leads to the facilitation of cell cycle progression and cell proliferation, and thus to the formation of lung tumors.

In addition to the upregulation of p21^CIP^ expression, we figure out that CDK2 activity was inhibited by p21^CIP1^, and the phosphorylation level of the CDK2 targeting site T821 of RB was less than the level in the control cells (Fig. 2e, f). Our data demonstrated a precise signal axis of p21^CIP1^ in the RAGE-mediated growth inhibition of adenocarcinoma cells. Moreover, the literature reports that p21^CIP1^ may be involved in the migration and invasion of cancer cells^29–31^. Despite our data revealing that p21^CIP1^ mediates RAGE-induced growth inhibition and cell cycle retardation, the possibility that p21^CIP1^ may participate in RAGE-promoted migration of lung adenocarcinoma cells is not excluded. Therefore, the role of p21^CIP1^ in RAGE-promoting migration remains to be further explored.

The downregulation of E-cadherin is one of the hallmarks of EMT in cancers. Snail, Slug, and Twist act as oncogenic transcription factors by suppressing E-cadherin expression^32–35^. In addition, ERK1/2 may upregulate these transcription factors to mediate cell migration, invasion, and EMT progression^1,36–38^. In the present study, the in vitro data showed that RAGE activated ERK1/2 to promote migration (Fig. 3d–f) while the proliferation and cell cycle progression were suppressed. Additionally, we provided evidence that RAGE-promoted migration and EMT were mediated through ERK1/2-induced activation of Snail, Slug, and Twist in lung adenocarcinoma cells (Fig. 3b–d). In addition to A549 cells, the lung adenocarcinoma cell lines CL1-0 and CL1-5 were also used to characterize the role of RAGE in cell growth and mobility (Fig. 4). CL1-0 is a poorly differentiated adenocarcinoma cell line isolated from a 64-year-old male patient, and the fifth sub-line CL1-5 with progression and invasiveness was selected by trans-well assay^18^. The CL1-5 cells were more mobile than CL1-0 cells, however their growth rate was slower than CL1-0 cells (Fig. S3). The level of RAGE protein in CL1-5 cells was more abundant than that in CL1-0 cells. In addition, the expression patterns of EMT markers, as well as p21^CIP1^, phosphorylated-ERK1/2, Snail, Slug, and Twist in CL1-5 cells, all represented the same tendency as RAGE-overexpressed A549 cells. Correspondingly, overexpressed RAGE in CL1-0 cells increased migration capacity and showed the same expression trends of the above markers as RAGE-overexpressed A549 cells. It implies that RAGE contributes to growth inhibition and EMT progression in lung adenocarcinoma cells.

The results analyzed from tumor xenograft mice showed an increasing number of metastatic nodules in lung in RAGE-overexpressed groups, implying that RAGE did contribute to the metastasis of lung adenocarcinoma (Fig. 5c). However, in the xenograft model, tumor growth in RAGE-overexpressed groups accelerated after the 5th week of tumor cell implantation, and the final average tumor size was larger than it of control group (Fig. 5b). RAGE-ligand interactions have been reported to mediate angiogenesis and cytokine secretion, thereby promoting cancer growth^39^. The result in Fig. 6c showed an increase expression of CD31, one of the endothelial markers, in inoculated tumors of RAGE-overexpressed groups. In addition, RAGE signals can influence the cross talk between cancer and immune system cells^40^. A literature indicated that RAGE activation through its ligand S100A7 may enhance tumorigenesis by recruiting TAMs in breast cancer^41^. In the present study, the in vivo experiments provided an evidence that RAGE may enhance the infiltration of TAMs to the tumor stroma (Fig. 6a, b). Therefore, the in vivo effect of RAGE signaling may provide a complicated TME beneficial for cancer growth, as well as metastasis.

The overall protein expression patterns of p21^CIP1^, p-ERK1/2, and RAGE in xenograft tumors also represent a consistent expression pattern with the in vitro data (Fig. 5f). However, in the comparison of the protein analysis from each individual mouse, the protein levels of p21^CIP1^ and p-ERK1/2 were not absolutely correlated with the level of RAGE. The RAGE-ligand interaction can be regulated by alternative spliced products; dominant negative RAGE (DN–RAGE), which lacks the cytosolic tail; and endogenous secretory RAGE (esRAGE or sRAGE), which is a soluble protein comprising only the extracellular region^42,43^, that acts as a decoy receptor to prevent the cellular responses mediated through RAGE signaling. However, the mechanism which gives rise to RAGE cleavage remains unclear. Understanding whether the individual differences of protein levels in xenograft tumors result from RAGE cleavage and identifying the mechanisms involved in this regulation require further study.

The RAGE-ligand signaling axis may promote the migration, invasion, and metastasis of a variety of cancers^5,14,44^. For example, the binding of RAGE and S100A8/A9 promotes the migration and invasion of human breast cancer cells^8^, and glucose-derived AGEs promotes the invasion and metastasis of gastric cancer through the activation of RAGE/ERK/Sp1/MMP2 pathway^45^. Moreover, expression of RAGE itself is also closely associated with the invasiveness and metastatic activities of gastric cancer^6^. Additionally, microRNA is reported to participate in RAGE-ligands mediated migration or cancer progression^46–48^. In this study, RAGE was overexpressed to hoist RAGE-mediated signaling and to investigate the effect of RAGE axis on the tumorigenesis of lung cancer. Although the literature has revealed a decreased expression of RAGE in lung tumors and suggested the down-regulation of RAGE supports lung carcinoma^9,11^, there is evidence implying that the RAGE-ligand interactions participate in the migration and invasion of lung cancer^6,14,15^. In addition, a recent study has reported that the down-regulation of RAGE by small interfering RNA (siRNA) inhibits migration and invasion through negatively regulating PI3K/AKT and KRAS/RAF-1 signaling cascades in lung cancer H1975 cells^49^. Moreover, a case report showed that a patient with primary systemic amyloid light-chain (AL) amyloidosis with early-stage non-small-cell lung cancer displayed strong expression of RAGE in tumor tissues, which suggests that an interaction between amyloid-containing tissues and RAGE-expressing cancer cells may progress both lung cancer and amyloidosis^50^.

The role of RAGE in the formation of lung cancer is still unclear, which implies its relevance and complexity in cancer research. In this study, we identified the dual role of RAGE, which might cause growth inhibition in the early of tumor formation while promoting EMT and providing a beneficial TME for tumorigenesis in lung adenocarcinoma. We provided the evidence with possible involvement of macrophage in lung tumor microenvironment to explain the contradictory findings of RAGE. This is the first study illustrating the facilitating role of RAGE on TAM recruitment in lung cancer and providing the reasonable answer to the controversial roles of RAGE in lung cancer growth with evidence.

**Fig. 7:**
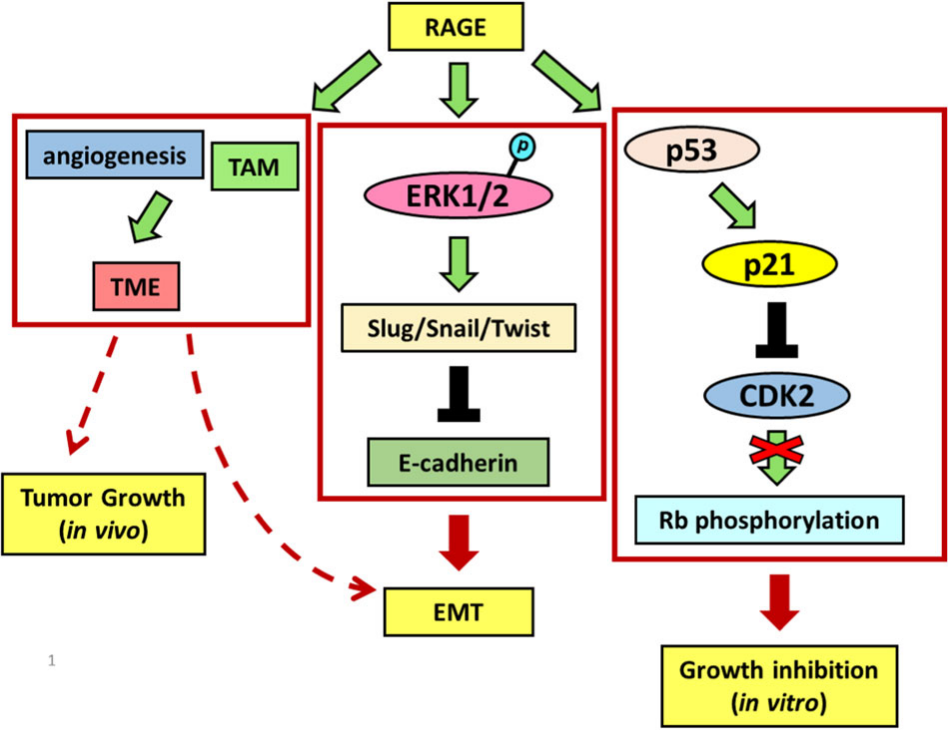
Schematic representation of RAGE function in lung adenocarcinoma cells. RAGE modulates cell growth by p53-dependent p21^CIP1^ up-regulation, which restrains the kinase activity of CDK2 and then blocks the complete phosphorylation of RB. Otherwise, RAGE promotes EMT progression through ERK activation, which decreases E-cadherin through up-regulates Slug, Snail, and Twist expressions. RAGE enhances angiogenesis and TAM infiltration, providing a beneficial tumor microenvironment for tumorigenesis.

In conclusion, RAGE possibly in cooperation with its ligands, might play an oncogenic role in contributing to the development of lung cancer (Fig. 7). These results not only shed light on the application of RAGE and its ligands in the future diagnosis and treatment of lung cancer, but also on the future investigation of RAGE in lung tumor microenvironment, which was neglected before.

## Materials and methods

### Cell culture

Human lung cancer A549 (purchased from the Bioresource Collection and Research Center, Taiwan) was used to perform cell line authentication (STR analysis in 2017 by Mission Biotech Co., Taiwan). CL1-0 and CL1-5^18^ cell lines were provided by Jeremy J.W. Chen, NCHU, Taiwan. All three cell lines were maintained in RPMI-1640 medium (Gibco/BRL, MD, USA), supplemented with 10% fetal bovine serum (FBS; Hyclone, Utah, USA), 2 mM l-glutamine, and 1 mM sodium pyruvate in a humidified atmosphere of 5% CO_2_ at 37 °C.

### Establishment of RAGE-overexpressed stable clones

An expression vector containing human RAGE (AGER) open reading frame (pUNO1-hAGER, InvivoGene, California, USA) or empty-vector (pUNO1-mcs) was delivered into A549 lung cancer cells by performing gene transfection methods according to the manufacturer’s instructions (jetPRIME^®^-Versatile in vitro DNA & siRNA transfection reagent, Polyplus-transfection, New York, USA). The stable clones were selected by continuously screening the above transfected cells with blasticidin (400 μg/ml, Sigma, USA) for 8 weeks. The protein level of RAGE was analyzed by Western blot analysis.

### Transwell in vitro migration assay

For in vitro the migration assay, 5 × 10^4^ cells suspended in serum-free (SF) medium were dropped into the hanging cell culture inserts (pore size: 8.0 µm; Merck Millipore, Darmstadt, Germany). FBS (10% in medium) was used as a chemoattractant in the lower compartment of the chamber. After incubation for 6 h the medium was aspirated, and the filters were washed with PBS and fixed with methanol before undergoing Giemsa staining. The migrated cells were counted in three random microscopic field images. All experiments were performed in triplicate and the data were shown as a mean ± SD.

### Kinetic cell migration assay

Wound healing assay was performed to evaluate kinetic migration. Cells were seeded into the silicone culture inserts (Ibidi, Munich, Germany) placed on the cell culture surface (µ-slide, Ibidi), and the inserts were removed after appropriate cell attachment, which created a 500-μm cell-free gap. Time-lapse images were recorded using a confocal microscope (FV1000, Olympus, Tokyo, Japan) with image processing software (FV10-ASW4.2, Olympus) to investigate cell migratory activity.

### Immunoprecipitation and western blot analysis

Cells were lysed with RIPA buffer (50 mM Tris-HCl, 150 mM NaCl, 1% Triton X-100, 0.25% sodium deoxycholate, 5 mM EDTA, 1 mM EGTA and supplemented with protease and phosphatase inhibitors), then centrifuged at 13,000 rpm for 30 min. Protein content was determined using the Bradford assay. Immunoprecipitates were collected by binding with magnetic beads (SureBeads, Bio-Rad Laboratories Inc., California, USA) in accordance with the manufacturer’s instructions. For Western blot analysis, equal amounts of protein samples were separated by sodium dodecyl sulfate polyacrylamide gel electrophoresis (SDS-PAGE) and blotted onto a polyvinylidene difluoride membrane (PVDF; Merck Millipore). After blocking with 5% skim milk, the primary antibody was used to react with the blots at 4 °C overnight. After that, the blots were incubated with horseradish peroxidase-labeled secondary antibody. Immuno-reactive bands were visualized using a Western Lightning™ Chemiluminescence Reagent Plus kit (Perkin Elmer LAS, Inc. Massachusetts, USA). The following primary antibodies were purchased commercially: RAGE (Merck Millipore), p21^CIP1^, p53, P-RB^S780^, P-RB^S795^ (Cell Signaling Technology, Danvers, MA, USA), ERK1/2, phosphorylated-ERK1/2 (P-ERK1/2) (Calbiochem, San Diego, CA, USA), P-RB^T821^ (Abcam, Cambridge, UK), CDK4, Vimentin (BD Biosciences, Franklin Lakes, New Jersey, USA), cyclinD1, CDK2, p27, RB, Snail, Slug, Twist, and β-actin (Santa Cruze Biotechnology, Dallas, Texas, USA).

### Real-time quantitative PCR

Quantitative real-time PCR was performed as described previously^51^. In brief, total RNA was extracted from cells using a Miniprep Purification Kit (Genemark, Taipei, Taiwan), and reverse transcription-PCR was performed with a high-capacity cDNA reverse transcription kit (Applied Biosystems, Foster City, CA, USA) according to the manufacturer’s protocol. The following primers were used to amplify the cDNAs: *CDKN1A* (p21^CIP1^) (5′-AAGATCTACTCCCCCATCAT-3′ and 5′-ACCCTAGTTCTACCTCAGGC-3′) and *ACTB* (β–actin) (5′-TTGCCGACAGGATGCAGAA-3′ and 5′-GCCGATCCACACGGAGTACT-3′). cDNA and primers were mixed within FastStart Universal SYBR Green Master (Roche Applied Science, Penzberg, Germany) and measured using a real-time PCR instrument (Applied Biosystems, Waltham, Massachusetts, USA). Data were presented using Ct values and adjusted relative to the levels of *ACTB* (β-actin) gene.

### In vitro kinase assay

The CDK2/CDK4 kinase assay was performed as described previously^52^. In brief, immunoprecipitates were incubated in kinase reaction buffer containing substrate histone H1 (Merck Millipore) and [γ-^32^P]-ATP (Perkin Elmer) or cold ATP (Sigma), comprising a final volume of 40 μl at 30 °C for 30 min. The level of phosphorylated histone H1 was identified using 10% SDS-polyacrylamide gel electrophoresis and visualized on the X-ray film (Fujifilm, Tokyo, Japan).

### Immunocytochemistry

Cells cultured on coverslips were fixed in 4% paraformaldehyde in PBS at room temperature. After fixation, cells were permeabilized with 0.3% Triton X-100 and 3% bovine serum albumin (BSA) in PBS and subsequently blocked in 3% BSA-PBS at room temperature. Anti-RAGE antibody (MAB5328, Merck Millipore) was used for immunoreaction, then hybridized with Alexa Fluor 488-conjugated goat anti-mouse 2nd antibody (ab150113, Abcam). After mounting, the images were investigated and recorded with confocal microscope.

### Small interfering RNA transfection

Cells were seeded in six-well plates and transfected with specific siRNA (p21 ^CIP1^ siRNA (si-CDKN1A): sc-29427; p53 siRNA: sc-29435, Santa Cruz Biotechnology) using jetPRIME^®^ transfection reagent (Polyplus-transfection) in accordance with the manufacturer’s instructions. After 24 h of incubation, the medium was replaced with complete medium, then cultured for a further 24 h before further analysis.

### The nude mice xenograft lung cancer model

Six-week-aged male nude mice (BALB/c nu/nu mice) were purchased from the National Laboratory Animal Center, National Science Council, Taiwan. The care and use of experimental animals complied with the ARRIVE guidelines and were all performed in accordance with protocols reviewed by the Institutional Animal Care and Use Committee (IACUC) of Taichung Veterans General Hospital, Taiwan (Approval numbers: La-1041303). Trypsinized and resuspended cells were mixed with Matrigel (BD Biosciences) at a 1:1 ratio (2.5 × 10^6^ cells/200 μl), then injected subcutaneously into the lower right flank of each mouse. A total of 24 mice were randomly assigned divided into three groups: parental control group, RAGE-overexpressed A549 subclone 1 group (RAGE^low^), and RAGE-overexpressed A549 subclone 2 group (RAGE^high^). There was no statistical difference in the mean of body weight of each group. Treatments in each group were blinded while IHC staining was performed.

Tumor volume was measured and calculated according to the formula L × W × W × *π*/6 (L: long axis; W: short axis) in units of mm^3^. The experiment was ceased in the 6th week (*n* = 3) and the 10th week (*n* = 5). Afterwards, the solid tumor tissues were dissected and sheared into fine pieces for protein extraction, followed by western blot analysis to detect protein levels of p-ERK/ERK1/2, p21^CIP1^, and RAGE. The expression and distribution profiles of RAGE and p21^CIP1^ in metastatic nodules in lung tissue were examined using immunohistochemistry (IHC).

### Statistical analysis

All data were represented as mean ± SD. Statistical analysis was performed using Student’s *t*-test for pairs with the following significance levels: *p < 0.05, **p < 0.01, and ***p < 0.001. All figures were generated from at least three repeated experiments with a similar pattern.

## Supplementary information

Supplementary Figure legends

Video 1. Time-lapse live cell image for wound healing assay of A549 parental cell line.

Video 2. Time-lapse live cell image for wound healing assay of A549 RAGE-overexpressed subclone.

Figure S1. The role of p21^CIP1^ in RAGE transiently overexpressed A549 cells.

Figure S2. The effect of RAGE on migration ability of A549 cells.

Figure S3. The comparison of cell growth between CL1-0 and CL1-5 cells.

Figure S4. The effects of RAGE on body weight in xenograft model.

Figure S5. The effects of RAGE on the metastatic lung nodules in xenograft model.
