# Supplementary material for: RAGE acts as an oncogenic role and promotes the metastasis of human lung cancer
Source: Cell Death Dis. 2020 Apr 23;11(4):265. doi: 10.1038/s41419-020-2432-1 (PMC7181650; doi:10.1038/s41419-020-2432-1)
Supplement: Supplementary file 1 — Supplementary Figure legends [file 41419_2020_2432_MOESM1_ESM.docx]

**Video 1. Time-lapse live cell image for wound healing assay of A549 parental cell line.** A549 parental cells were seeding and attached on cell culture surface (u-slide, ibidi, Munich, Germany) with a 500 μm cell-free gap and maintained in serum-free culture medium for 24 h. A fixed-point image was taken every 15 min by a confocal microscope for continuous shooting to record a time-lapse image.

**Video 2. Time-lapse live cell image for wound healing assay of A549 RAGE-overexpressed subclone.** A549 RAGE-overexpressed cells were seeding and attached on cell culture surface (u-slide, ibidi, Munich, Germany) with a 500 μm cell-free gap and maintained in serum-free culture medium for 24 h. A fixed-point image was taken every 15 min by a confocal microscope for continuous shooting to record a time-lapse image.

**Figure S1. The role of p21^CIP1^ in RAGE transiently overexpressed A549 cells.** A549 cells were co-transfected with RAGE plasmid and siRNA against CDKN1A (p21^CIP1^) or control siRNA for 24 h then trypsinized and seeded into 24-well plates. After cultured for 24 h, cells were harvested to evaluate the protein levels of RAGE and p21^CIP1^ by western blotting (a), and cell growth was measured to evaluate the effect of p21^CIP1^ knockdown on cell growth in A549 cells with transiently overexpressing RAGE (b). *indicates p < 0.05 compared to the si-control group. ++ indicates +++ indicates p < 0.001 compared to RAGE plasmid with si-control group.

**Figure S2. The effect of RAGE on migration ability of A549 cells.** The migration ability was evaluated by wound healing assay. The images showed the selection of tracks of cell movement corresponding to the distance moved by the cells within 12 h of time-lapse imaging.

**Figure S3.** **The comparison of cell growth between CL1-0 and CL1-5 cells.** Cells were seeded into 24-well culture plates (5 x 10^3^ cells/well) and cell number was recorded every 24 h within 5 days.

**Figure S4.** **The effects of RAGE on body weight in xenograft model.** A total of 24 mice were divided into three groups. Parental or RAGE overexpressed A549 cells were injected subcutaneously into the right low flank of mice. The body weight was recorded once a week.

**Figure S5. The effects of RAGE on the metastatic lung nodules in xenograft model.** Mice were divided into three groups, and then A549 subclones were injected subcutaneously into the right low flank of mice. The experiment was ceased in week 6^th^ (n=3) and week 10^th^ (n=5). The lungs were collected and perfused with PBS then fixed with formalin. The visible lung metastases nodules were counted as indicated by the white arrows.
