# Supplementary figures and images for: RAGE acts as an oncogenic role and promotes the metastasis of human lung cancer
Source: Cell Death Dis. 2020 Apr 23;11(4):265. doi: 10.1038/s41419-020-2432-1 (PMC7181650; doi:10.1038/s41419-020-2432-1)

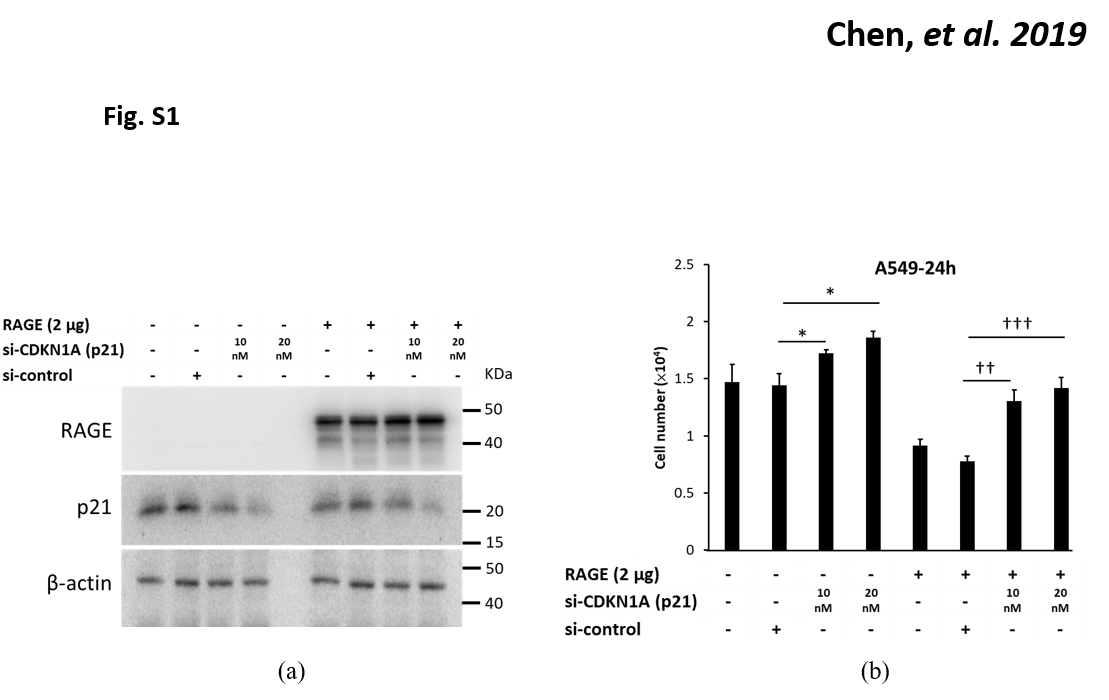

Supplement: Supplementary file 4 — Figure S1. The role of p21CIP1 in RAGE transiently overexpressed A549 cells. [file 41419_2020_2432_MOESM4_ESM.png]

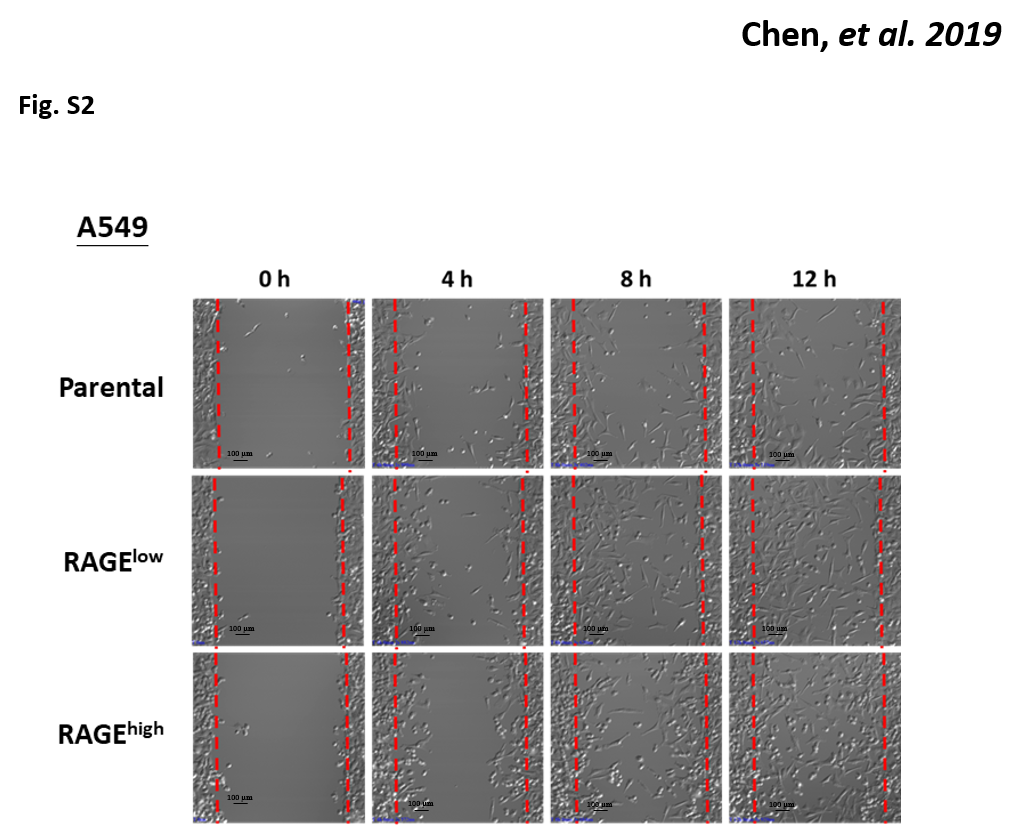

Supplement: Supplementary file 5 — Figure S2. The effect of RAGE on migration ability of A549 cells. [file 41419_2020_2432_MOESM5_ESM.png]

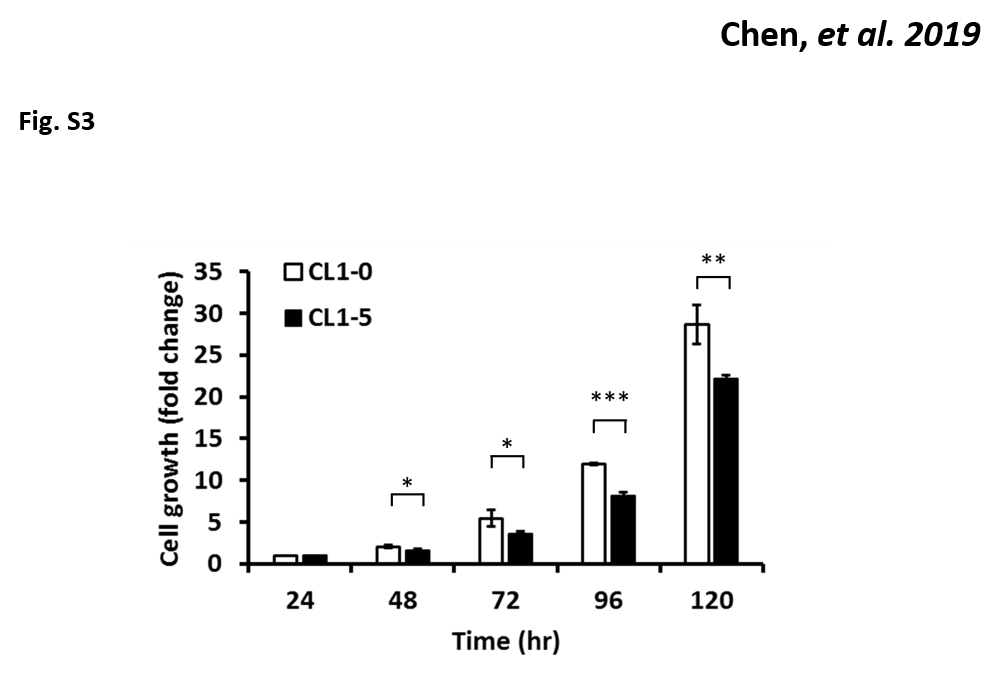

Supplement: Supplementary file 6 — Figure S3. The comparison of cell growth between CL1-0 and CL1-5 cells. [file 41419_2020_2432_MOESM6_ESM.png]

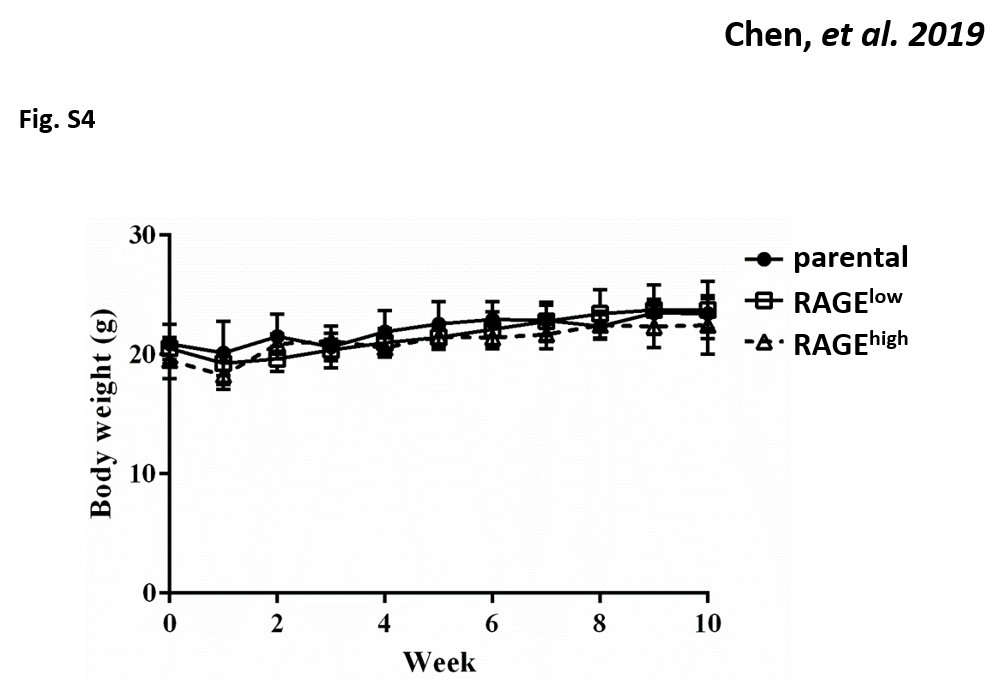

Supplement: Supplementary file 7 — Figure S4. The effects of RAGE on body weight in xenograft model. [file 41419_2020_2432_MOESM7_ESM.png]

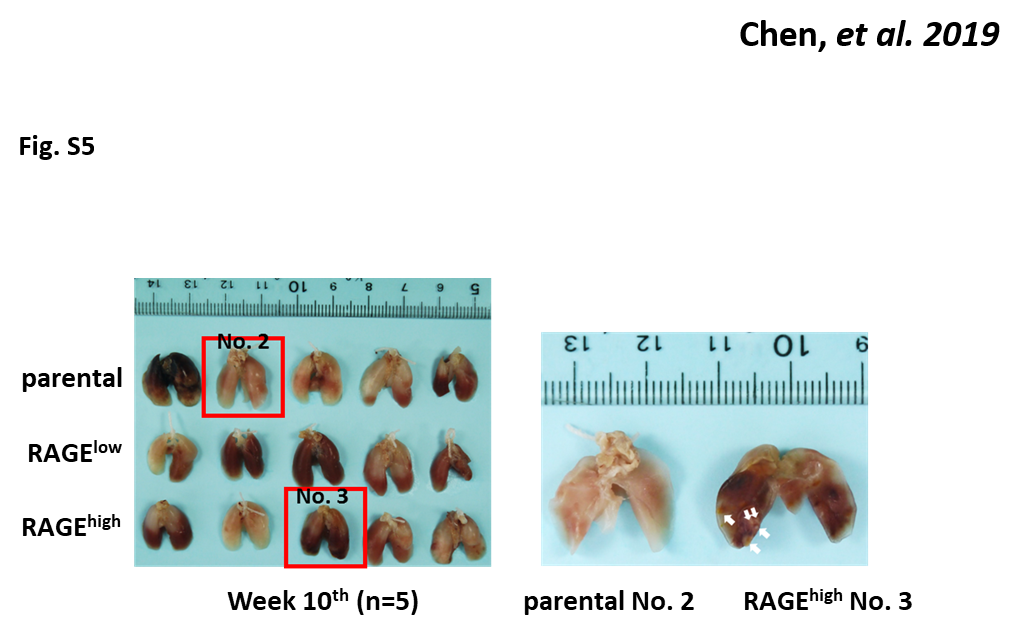

Supplement: Supplementary file 8 — Figure S5. The effects of RAGE on the metastatic lung nodules in xenograft model. [file 41419_2020_2432_MOESM8_ESM.png]
